# Evaluation of a peer education program on student leaders’ energy balance-related behaviors

**DOI:** 10.1186/s12889-017-4707-8

**Published:** 2017-09-07

**Authors:** B. C. Foley, V. A. Shrewsbury, L. L. Hardy, V. M. Flood, K. Byth, S. Shah

**Affiliations:** 1 Western Sydney Local Health District, Westmead, NSW Australia; 2 Sydney Medical School, The University of Sydney, Sydney, NSW Australia; 3 Faculty of Health Sciences, The University of Sydney, Sydney, NSW Australia

**Keywords:** Adolescents, Peer education, Secondary schools, Energy balance-related behavior, Intervention

## Abstract

**Background:**

Few studies have reported energy balance-related behavior (EBRB) change for peer leaders delivering health promotion programs to younger students in secondary schools. Our study assessed the impact of the Students As LifeStyle Activists (SALSA) program on SALSA peer leaders’ EBRBs, and their intentions regarding these behaviors.

**Methods:**

We used a pre–post study design to assess changes in EBRBs and intentions of Year 10 secondary school students (15–16 year olds) who volunteered to be peer leaders to deliver the SALSA program to Year 8 students (13–14 year olds). This research is part of a larger study conducted during 2014 and 2015 in 23 secondary schools in Sydney, Australia. We used an online questionnaire before and after program participation to assess Year 10 peer leaders’ fruit and vegetable intake, daily breakfast eating, sugar sweetened beverage (SSB) intake, moderate-to-vigorous physical activity (MVPA) participation and school-day recreational screen time behaviors and intentions regarding these EBRBs. Generalized estimating equations with a robust variance structure and exchangeable correlation structure were used to estimate the individual-level summary statistics and their 95% CIs, adjusted for clustering. We further assessed the effect of covariates on EBRB changes.

**Results:**

There were significant increases in the proportion of Year 10 peer leaders (*n* = 415) who reported eating ≥2 serves fruit/day fruit from 54 to 63% (*P* < 0.01); eating ≥5 serves vegetables/day from 8 to 12% (*P* < 0.01); and drinking <1 cup/day of SSBs from 56 to 62% (*P* < 0.01). Change in ≥60 min MVPA participation/day depended on gender (*P* < 0.01): Boys increased 14% while girls decreased −2%. Changes in eating breakfast daily also depended on gender (*P* < 0.004): Boys increased 13% while girls decreased −0.4%. The change in peer leaders recreational screen time differed by socio-economic status (*P* < 0.05): above average communities decreased by −2.9% while below average communities increased 6.0%. Significant shifts were seen in peer leaders’ intentions, except MVPA which remained stable.

**Conclusions:**

The SALSA program had a positive impact on peer leaders’ EBRBs, with gender and socio-economic status moderating some outcomes.

**Trial registration:**

ACTRN12617000712303 retrospectively registered.

**Electronic supplementary material:**

The online version of this article (10.1186/s12889-017-4707-8) contains supplementary material, which is available to authorized users.

## Background

Non-communicable diseases (NCDs), such as cardiovascular disease, type 2 diabetes, obesity and cancer, cost health care systems billions of dollars annually and are associated with significant morbidity and premature mortality worldwide [1]. Many risk factors related to these diseases, including energy balance-related behaviors (EBRBs) such as poor nutrition and insufficient physical activity, originate during adolescence and track into adulthood [2]. Over one-quarter of adolescents in Australia are overweight or obese, the highest level in decades. Evidence shows the gap in unhealthy weight status is widening between adolescents from low and high socioeconomic neighborhoods, with little improvement seen in those most in need [3]. Almost 60% of adolescents do not meet recommendations for vegetable intake, recreational screen-time or physical activity, and adolescents are the largest consumers of ‘junk’ foods [3, 4]. This pattern of behavior is consistent globally among adolescents and there is a need to develop more effective NCD prevention interventions for this age group.

Adolescence is a period of transition during which young people become less dependent on their family and spend more time with their peers. During secondary schooling the importance of peer relationships intensifies [2, 5]. It is recognized that peers have a greater influence on health behaviors of adolescents than parents, teachers or health professionals [6, 7]. Peer-led initiatives are seen as a salient characteristic of promising interventions in secondary schools to improve adolescents’ EBRBs, yet little research has been done with 15–16 year olds [8, 9].

Since 2004, the peer-led Students As LifeStyle Activists (SALSA) program has evolved, through a partnership between health and education sectors, to improve dietary behaviors and reduce physical inactivity in secondary school students. Our peer education model has been adapted from an intervention effective at improving asthma outcomes in adolescents in Australia and Jordan [10, 11]. The SALSA program is based on notions of modelling, self-efficacy, peer pressure and environment from Bandura’s social cognitive theory, and Freire’s empowerment education approach; and aligns with the World Health Organization’s Health Promoting Schools Framework [12–15]. In the SALSA program, the primary target group is Year 8 secondary school students (13–14 year olds). They receive four SALSA lessons on healthy living led by Year 10 students (15–16 year olds) who have been trained as SALSA peer leaders in a one day workshop [13, 14]. It is essential that peer leaders are well-trained to achieve program implementation integrity [16, 17]. During the SALSA program, students are encouraged to observe and imitate the behaviors of their peers, increasing their self efficacy in implementing new skills and gain positive attitudes about health [15]. The key role of a peer leader is to model behaviors, facilitate group processes, encourage inquiry, critical thinking and reasoning skills in younger students [16].

Pearlman et al. has demonstrated the influence of peer education on knowledge and behaviors for both students and peer leaders through a communicable disease prevention program [18]. To date the majority of studies on the impacts of peer education prevention programs for NCD have reported the effects on the target (youngest) group. Some programs have shown significant improvements in younger students’ diet quality, self-efficacy, and knowledge of healthy living, but the effect on peer leaders is less clear [10, 19–21]. Evaluations of school-based peer education for NCD prevention programs have previously been limited to feasibility and acceptability for peer leaders delivering peer education [15, 22]. The purpose of our study was to assess the impact of the SALSA program on Year 10 SALSA peer leaders’ dietary, physical activity and recreational screen time behaviors, and their intentions regarding these EBRBs. We hypothesize that peer leaders’ EBRB may improve following their participation in the SALSA program.

## Methods

### Aim, design, setting

This research is part of a larger study on the impact of a peer education intervention (the SALSA program) on EBRBs of secondary school students. We used a pre–post study design to investigate the impact of the SALSA program on Year 10 peer leaders’ EBRBs and intentions regarding these behaviors across 23 participating secondary schools in Western Sydney, Australia. The pre-post study involved outcome measurement of participants immediately before the peer leader training workshop (Before SALSA) and two-weeks after they had delivered the final SALSA lesson to Year 8 students (After SALSA).

### Participants

The SALSA program was offered to all secondary schools (*n* = 88) in the Western Sydney region. We recruited schools for this study during 2014 and 2015 until the recruitment aim (*n* = 23) was achieved. At participating schools, Year 10 peer leaders either volunteered or were selected by teachers to be trained to deliver the SALSA program to their Year 8 peers. The peer leader training workshop was delivered by volunteer university students (SALSA educators) from health and education faculties who had received SALSA educator training from project staff.

Ethics approval for this study was granted from the University of Sydney Human Research Ethics Committee (Approval no: 2014/203), the NSW Department of Education and Training (State Education Research Application Process no: 2,014,096) and the NSW Catholic Education Commission. Written consent was not required. Adolescents or their families could decline participation through an opt-out process.

### Intervention

The SALSA program is a peer-led, school-based educational program designed to motivate secondary school students to improve their food, beverage, physical activity and recreational screen time behaviors. Each participating school appoints a teacher to coordinate the following intervention components in conjunction with SALSA project staff.

#### Peer leader training workshop

University students, who had been trained as SALSA educators, visited participating secondary schools to deliver a one-day SALSA peer leader training workshop to Year 10 students. During the training workshop, SALSA educators explained and practiced all activities within the structured Year 8 SALSA lessons while Year 10 students actively participated. The aim of the workshop was to train Year 10 peer leaders to successfully deliver the SALSA lessons to Year 8 students. To complete their training, peer leaders were provided with a scripted manual to use as a guide and given the opportunity to practice delivery of the lesson content in groups in front of their peers and educators (see Additional file 1). Feedback was provided after the practice from SALSA educators to develop peer leaders’ educator skills.

#### Year 8 SALSA lessons

The SALSA lessons cover healthy eating, physical activity and living a healthy lifestyle using alternative methods of learning including a video, games, role-playing and a quiz show [15]. The four, 70-min SALSA lessons are designed to integrate with the Personal Health, Development and Physical Education (PDHPE) curriculum. The school SALSA coordinator allocated trained peer leaders to deliver the SALSA lessons in groups. We recommended between four and six peer leaders per group. Each group of peer leaders delivered the four SALSA lessons to 1 Year 8 PDHPE class. The Year 8 PDHPE teacher provided general supervision and only assisted with behavior management as needed.

### Outcome measures

Online self-report questionnaires were used to assess Year 10 peer leaders’ dietary behaviors, physical activity, screen time and intentions to change these behaviors. Dietary questions were based on validated questions from a short food frequency questionnaire and included daily intake of fruits, vegetables, drinking of sugar sweetened beverages (SSBs) and frequency of eating breakfast [23–25]. Participation in physical activity and week day screen time outside of school hours were measured using validated questions [26, 27]. Three additional questions were included in the online questionnaire completed after the SALSA program: “Would you recommend the SALSA program to other high school students?”, “What was the most important message from the SALSA Program?” and “Have you talked about the SALSA Program with your family? If yes, what did you talk about?”.

#### Covariates

The baseline online questionnaire asked peer leaders to report their gender and the primary language spoken at home. Peer leaders were assigned their school’s Index of Community Socio-Educational Advantage (ICSEA) as an indicator of school-level socio-economic status [28].

#### Process evaluation

Year 8 classroom teachers were asked to keep a paper-based lesson log to record the SALSA lesson delivery dates, the number of peer leaders and Year 8 students who participated in each lesson. This lesson log was returned to the study team after all SALSA lessons had been completed.

### Statistical analysis

We analyzed our outcome data using the Statistical Package for the Social Sciences (SPSS) version 22.0. Generalized estimating equations with a robust variance structure and exchangeable correlation structure were used to estimate the individual-level summary statistics and their 95% confidence intervals (CIs) adjusted for clustering [29–31]. ICSEA score, as a proxy for socio-economic status, was categorized as low if it was below the Australian mean of 1000, and high if it was 1000 or more [28]. Peer leaders’ responses were dichotomized as ‘meeting’ or ‘not meeting’ Australian guidelines for adolescents’ daily fruit and vegetable intake, recreational screen-time, and MVPA [32, 33]. Further to this, we assessed whether peer leaders did or did not drink less than one cup of SSB per day and whether they ate breakfast daily. Qualitative responses were reviewed by two project staff to identify themes and then coded independently, according to the main themes. Themes were identified based on grounded theory*,* which is consistent with a framework analysis approach [34, 35].

## Results

We trained 96 volunteer university students as SALSA educators, who cumulatively coached 519 Year 10 students as SALSA peer leaders across 23 participating secondary schools. One participating secondary school was unable to complete the post-SALSA questionnaire after delivering the SALSA program due to extenuating circumstances at the school. Pre and post evaluation data were available for 415 (77%) Year 10 peer leaders (64% girls) who delivered the SALSA program to over 3800 Year 8 students at 22 secondary schools. Sixteen out of the 22 schools had a school-level ICSEA that was below the national average, which represents 60% of Year 10 peer leaders from communities of disadvantage. The most common language spoken at home was English (70%), while one-in-five spoke an Asian language (19%), and the remaining students spoke Middle Eastern (6%) or other (5%) languages. Seventeen out of 22 schools (77%) provided completed lesson logs. On average 4.5 (SD ± 2.6) Year 10 peer leaders delivered the lessons to each Year 8 class; which was a ratio of approximately 1 Year 10 peer leader per 5 Year eight students. The mean number of days between the first and final (fourth) SALSA lesson was 25 days (SD ± 15.9). Peer leaders at all schools delivered the four SALSA lessons as planned except one school omitted playing the video which is a key program resource.

Table 1 shows the changes in the proportion of Year 10 peer leaders who reported meeting EBRB recommendations before and after participating in the SALSA program. At follow-up, significant positive changes were observed in the proportion of adolescents meeting the recommendations for daily intake of fruits (8.8% increase) and vegetables (5% increase). The proportion of peer leaders who reported that they consumed less than one cup of SSB increased by 6.4%. There were no statistically significant changes in the proportion of peer leaders who ate breakfast daily, or who met recommendations for MVPA and recreational screen-time.

**Table 1:** Change in Year 10 peer leaders meeting energy balance-related behavior recommendations (**%,** 95% CI; *n* = 415)

Behavior	Recommendation [32, 33]	Before SALSA (%, 95%CI)	After SALSA (%, 95%CI)	Change^b^ (%, 95%CI)	*P value*
Fruit	≥2 serves/day	53.9 (47.1–60.4)	63.0 (58.0–67.8)	8.8 (3.4–14.2)	<0.01^a^
Vegetable	≥5 serves/day	7.5 (5.3–10.6)	12.2 (9.6–15.3)	5.0 (1.4–8.7)	<0.01^a^
Breakfast	Everyday	48.8 (41.6–56.2)	53.8 (45.8–61.7)	5.3 (−0.1 to 10.7)	0.053
Sugar-sweetened beverages	<1 cup/day	55.9 (48.8–62.9)	62.4 (53.0–70.9)	6.4 (1.5–11.2)	<0.01^a^
Screen-time	≤ 2 h/day	41.9 (36.7–47.2)	44.4 (38.0–51.0)	1.4 (−3.8 to 6.6)	0.592
MVPA	≥ 60 min/day	12.9 (8.5–19.0)	17.1 (12.7–22.7)	3.4 (−0.4 to 7.3)	0.076

^a^Statistically significant improvement (*P* < 0.05). Screen time = Recreational screen time on school days. MVPA = moderate-to-vigorous physical activity. ^b^Due to a strong school-level clustering effect, the % change may differ from expected

Table 2 shows the change in EBRBs stratified by gender. There were significant changes in the proportion of boys who met recommendations for fruit intake (12.9% increase), MVPA (14.2% increase), recreational screen-time (5.0% increase), and a 13.3% increase in the proportion of boys eating breakfast daily. Vegetable and SSB intake did not significantly change in boys after participating in the SALSA program. Among girls, there was a 7.2% increase in the proportion who met the recommendation for fruit intake and a 9.9% increase in the proportion who consumed less than one cup of SSB daily. The remaining outcomes measured did not change significantly in girls. When boys and girls were compared with each other, eating breakfast daily and meeting the MVPA recommendation were significantly different (Table 2).

**Table 2:** Change in Year 10 peer leaders meeting energy balance-related behavior recommendations, by gender (% 95%CIs)

Behavior	Recommendation [32, 33]	Boys (*n* = 148)			Girls (*n* = 267)			
		Before SALSA (%, 95% CI)	After SALSA (%, 95%CI)	Change (%, 95%CI)	Before SALSA (%, 95%CI)	After SALSA (%, 95%CI)	Change (%, 95%CI)	P* (between gender)
Fruit	≥2 serves/day	49.0% (42.1–55.9)	61.9% (55.0–68.4)	12.9% (3.7–22.1)^a^	55.3% (47.4–63.0)	63.7% (58.0–69.0)	7.2% (0.6–13.7)^a^	0.329
Vegetable	≥5 serves/day	8.1% (4.5–14.1)	14.3% (10.2–19.7)	4.2% (−0.1 to 8.5)	7.2% (4.6–11.1)	10.4% (6.9–15.5)	3.1% (−2.0 to 8.2)	0.590
Breakfast	Everyday	49.7% (38.9–59.4)	60.9% (49.3–71.4)	13.3% (3.4–23.2)^a^	47.1% (38.5–55.8)	49.1% (40.2–58.0)	−0.4% (−7.0 to 6.2)	0.004^a^
Sugar-sweetened beverage	<1 cup/day	48.5% (39.1–58.0)	48.9% (37.0–60.9)	1.5% (−7.4 to 10.5)	60.1% (52.5–67.2)	69.3% (59.7–77.5)	9.9% (3.9–15.8)^a^	0.139
Screen-time	≤2 h/day	48.3% (21.0–55.7)	51.5% (44.6–58.2)	5.0% (3.6–6.4)^a^	38.1% (32.5–44.1)	39.4% (31.7–47.7)	0.7% (−6.8 to 8.2)	0.558
MVPA	≥60 min/day	13.9% (8.6–21.8)	27.0% (18.8–37.1)	14.2% (6.9–21.5)^a^	12.6% (8.0–19.2)	10.2% (6.2–16.5)	−1.7% (−5.6 to 2.2)	<0.001^a^

^a^Statistically significant improvement (*P* < 0.05). MVPA = moderate-to-vigorous physical exercise. Screen time = recreational screen time on school days. P* Interaction between boys and girls

The change in peer leaders meeting recreational screen time recommendations differed by socio-economic status (*P* < 0.05). The proportion of peer leaders decreased by −2.9% (CI –3.4 to −2.4) at schools with an above average ICSEA, compared to those from schools with a below average ICSEA who improved school-day recreational screen time by 6.0% (CI –1.2 to 13.2). No other outcomes were moderated by socio-economic status or language spoken at home as covariates.

Table 3 shows Year 10 peer leaders’ intentions regarding EBRBs before and after SALSA program participation. After the participating in the SALSA program, a higher proportion of peer leaders’ intentions were consistent with recommendations for fruit intake, vegetable intake, recreational screen time on school days and guidance to eat breakfast every day. The proportion of students who intended to be physically active on all or most days of the week did not significantly change. No questionnaire item collected students’ intentions regarding SSB intake.

**Table 3:** Change in Year 10 peer leaders’ intentions for the next month regarding energy balance-related behaviors (%, 95% CI; *n* = 415)

Intentions	Before SALSA (%, 95%CI)	After SALSA (%, 95%CI)	Change^b^ (%, 95%CI)	*P value*
To eat ≥2 fruit serves/day	72.6 (66.1–78.2)	81.5 (77.2–85.2)	8.5 (2.9–11.4) ^a^	<0.01^a^
To eat ≥5 vegetable serves/day	16.9 (13.3–21.3)	29.5 (25.6–33.6)	15.1 (11.8–18.4) ^a^	<0.01^a^
To eat breakfast daily	67.6 (60.9–73.7)	77.1 (70.2–82.8)	8.3 (5.1–11.5) ^a^	<0.01^a^
To reduce recreational screen-time	36.6 (32.5–40.9)	45.7 (39.9–51.6)	9.7 (3.2–16.1) ^a^	<0.01^a^
To be more physically active	79.5 (75.1–83.3)	83.1 (79.1–86.5)	3.4 (−0.8–7.5)	0.110

^a^Statistically significant improvement (*P* < 0.05). ^b^Due to a strong school-level clustering effect, the % change may differ from expected

Ninety-one percent of peer leaders reported they would recommend the SALSA program to other high school students. The important messages of the SALSA program identified by peer leaders after participating in the program were categorized into the following emergent themes; overall health benefits of living a healthy lifestyle, healthy eating, being physically active, reducing recreational screen time, setting goals, leadership and teaching. Furthermore, 42% of peer leaders reported discussing similar themes with their families.

## Discussion

Our results indicate that secondary school students who volunteered to be peer leaders in the SALSA program improved their EBRBs and intentions to live a healthy lifestyle. Participation in the program had a positive impact on the proportion of peer leaders who met national recommendations for fruit and vegetable intake and who consumed <1cup SSBs/day [32, 33]. Additionally, there were significant increases in the proportion of peer leaders who reported intentions to increase their fruit and vegetable intake, to eat breakfast daily and to reduce recreational screen time. These results are promising, given that the SALSA program is a relatively low-intensity (a 1-day workshop for peer leaders and delivery of four 70-min SALSA lessons) and low cost program (primarily delivered by volunteers) [15, 17]. We have demonstrated that in the short term, the SALSA program holds benefits for peer leaders who were trained and delivered the school-based SALSA program to younger students.

Intentions to make positive changes to behavior are theorized to be an important consideration of the behavior change process but data on adolescent intentions are rarely reported in intervention studies [36]. There is some evidence that among adolescents, dietary and physical activity intentions are associated with behaviors [37–39]. Our measurement of intentions allows preliminary insight into the relationship between intentions and behavior in adolescents where most measured intentions were consistent with changes in reported behavior. Peer leaders intentions to eat breakfast daily and to reduce recreational screen time both improved after the SALSA program however this did not result in significant behavior change in the short term. The disparity between intent and action highlights the need for a multi-level approach to change behavior over time in environments that promote energy balance among adolescents.

Our covariate analysis revealed significant differences between boys’ and girls’ reported change in EBRBs. Boys showed greater improvements in the proportion eating breakfast daily and the proportion participating in MVPA. This is consistent with previous studies which have shown that girls are more likely to skip breakfast, often in an attempt to minimize weight gain [40, 41]. Complementary interventions to increase breakfast eating in peer leaders should be explored, especially those targeted at girls. The lack of change in girls’ physical activity after the SALSA program also requires further attention. Young girls have previously been identified as a priority population for physical activity interventions due to their low participation rates [41–43]. We expect that whole of school environmental changes and/or additional positive social marketing may be required to nudge girls towards the significant improvements seen in peer leader boys in our study.

Additionally, ICSEA influenced an increase in the proportion of peer leaders exceeding the recreational screen time guideline from schools with an above average ICSEA, compared to peer leaders in schools with a below average ICSEA. This was an unexpected change. In the current age, with increased use of mobile technologies, separating “recreational” and “educational” screen time is challenging to report. Our results show that more than half of 15–16 year olds in secondary schools exceeded the recommendation of less than 2 h per day. The interplay of screen time on health is an area of growing concern. Future evaluations should consider novel approaches to measuring screen time and its impact on health and wellbeing.

The SALSA peer leaders in our study were all participating on a voluntary basis, whether they volunteered or were selected by their teachers. Hence the participants in this study represent a select group. In a similar study, the TEENS intervention reports recruiting peer leaders through a classroom vote [21, 22]. This resulted in respected and admired students being selected for the role [21, 22]. Our peer education model aims to reduce health disparities by building capacity in any Year 10 student, rather than elevating those who are already highly regarded by their peers. The comprehensive peer leader training workshop, provides a solid basis for SALSA peer leaders to be successful in their role [17]. Teaching skills, as well as the critical appraisal, goal setting, communication and collaboration techniques gained by peer leaders in delivering the SALSA program, will be beneficial following their completion of secondary education. Furthermore, we awarded all SALSA peer leaders with a certificate to aid future employment applications. No other incentives for participation were provided. This is different to other peer education programs that have reported providing monetary incentives to their peer educators [44, 45].

It is possible that the reported changes in behavior were the result of the peer leader workshop, delivered by trained university students. However the SALSA program critically involves both peer leader training and then delivery of the program in secondary schools. We evaluated the cumulative influence of both processes on EBRBs which together contribute to re-enforcing the healthy lifestyle messages. We did not investigate the effects of one process versus the other as both processes combined are what underpins the peer education intervention model.

A strength of this study is that we recruited a socio-demographically diverse sample of Year 10 students who were trained to successfully deliver the SALSA program to their younger peers. Almost all peer leaders (91%) would recommend the SALSA program to their peers. This suggests the SALSA program continues to be relevant, age-appropriate and engaging for 15–16 year olds. Our high questionnaire completion rate (77%) at pre and post was achieved through the use of an opt-out consent process.

We were encouraged to find that 42% of peer leaders had talked about the SALSA program with their families. Peer leaders reported making changes at home, such as “I talked about how we should eat more healthy dinners and the benefits of having breakfast” (Year 10 boy) “How to change 1 or 2 things in a meal to make it healthier” (Year 10 girl) and “I told them how to be healthy and started taking action in my house” (Year 10 girl). This shows promise for adolescent-led changes in the home environment to promote healthy eating and physical activity. We will investigate how SALSA peer leaders can enhance their impact in their homes in future.

Our analysis adjusted the outcome measures for clustering effects of schools, however we did not audit the school environment for barriers and facilitators to eat well and be active at school. As a result, we are unable to report on student-led changes in the school environment which may have resulted from the school action plan developed in lesson four of the SALSA program. Future studies should assess changes in the school environment which will likely help sustain the short term behavior changes reported in our evaluation [46].

The use of a quasi-experimental study design was a limitation however in studies where randomization is not feasible, pre-post study designs are acceptable as they can be conducted at relatively low cost and increase the relevance of the findings for real world application [47]. In this study, it was not logistically possible to randomize schools or individual students. As a result, it is unclear if the changes observed were due to our intervention. A further limitation was the lack of longer term follow-up to determine the longevity of lifestyle behavior changes among SALSA peer leaders. Our outcome measures were identified as indicators of health and wellbeing across the life course and which have a strong influence on prevention of NCDs [2, 5]. The use a more detailed dietary assessment method (e.g. multi-day weighed food records or 24 h recalls) and objective measures of physical activity participation would have strengthened our study but was not logistically feasible. To assess whether the observed behavior changes are sustained, we will undertake further research to examine the long-term effectiveness of the SALSA program.

The peer leaders in our study were either 15 or 16 years old, an age group which is largely underserved by health promotion programs [2]. Our results support the assumption that training and subsequent education of peers influences EBRBs of peer leaders. This contributes towards a greater understanding of the effect of peer education programs on peer leaders’ EBRBs, associated with NCD prevention.

## Conclusions

Our evaluation of the SALSA program demonstrates that a school-based peer education program can positively impact energy balance-related behaviors of teenage peer leaders. Gender and socio-economic status had moderating effects on the reported behavior change after 2 weeks. Long term impacts of peer education programs on peer leaders energy balance-related behaviors and potential student-led environmental changes should be undertaken in future.

## Additional file

Additional file 1: The SALSA program peer leader manual. (PDF 11258 kb)

## Abbreviations

CIs: Confidence intervals
EBRB: Energy balance-related behavior
ICSEA: Index of community socio-educational advantage
MVPA: Moderate to vigorous physical activity
NCD: Non-communicable diseases
SALSA: Students As LifeStyle Activists
SSB: Sugar-sweetened beverages
