# Supplementary material for: Evaluation of a peer education program on student leaders’ energy balance-related behaviors
Source: BMC Public Health. 2017 Sep 7;17:695. doi: 10.1186/s12889-017-4707-8 (PMC5590169; doi:10.1186/s12889-017-4707-8)
Supplement: Additional file 1: — The SALSA program peer leader manual. (PDF 11258 kb) [file 12889_2017_4707_MOESM1_ESM.pdf]

STUDENTS AS LIFE STYLE ACTIVISTS

SALSA

Peer Leader Manual

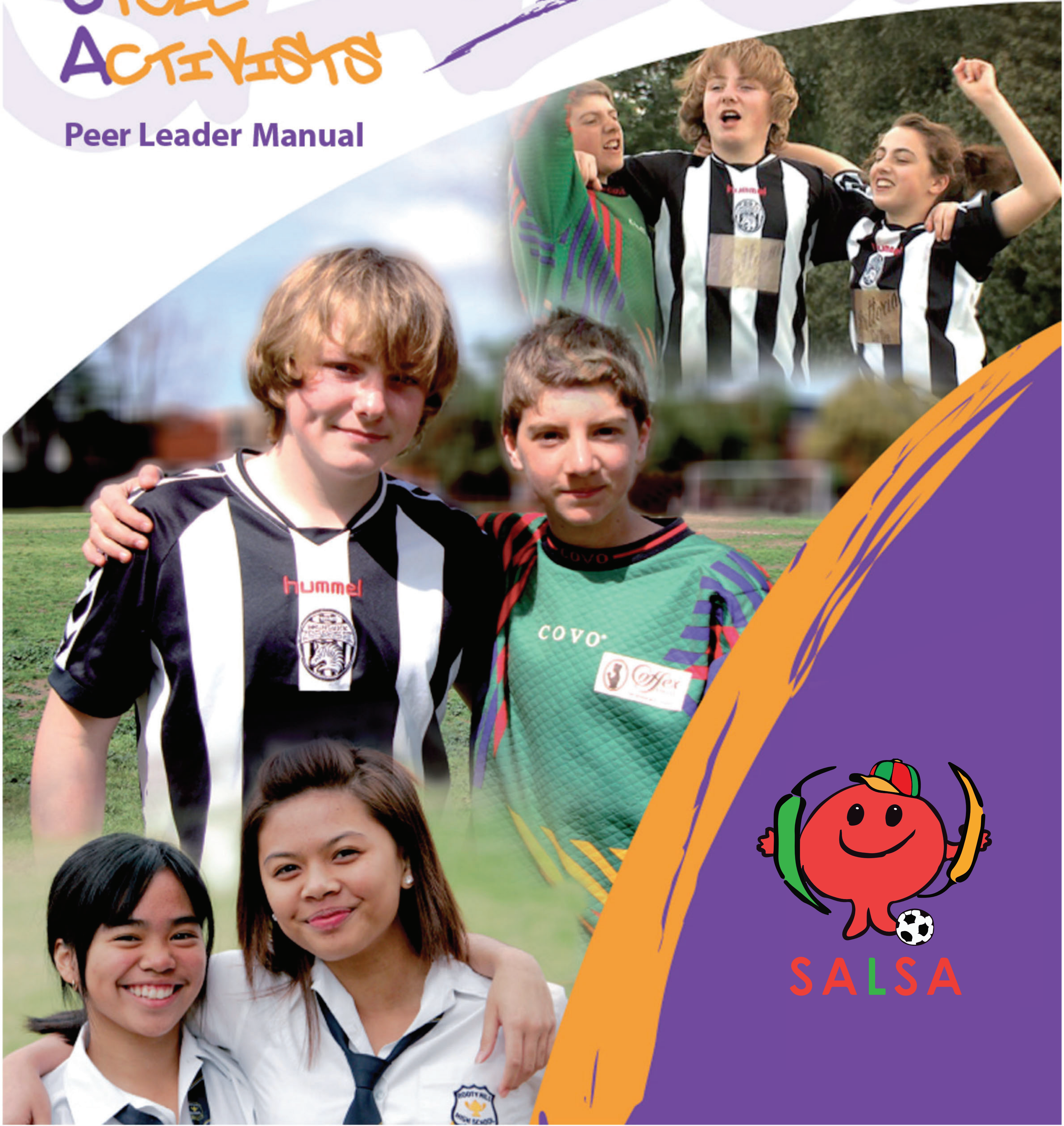

SALSA

SALSA

SALSA Peer Leader Manual

Students As LifeStyle Activists (SALSA) is a unique peer educational program designed to motivate high school students to increase physical activity, improve diet and lead a healthier lifestyle. The SALSA Peer Leader Manual is a component of the SALSA Kit, which was developed and tested as part of a collaboration formed between health and education within Western Sydney.

Suggested citation: Shah S, Andrew A, Hillery C, Pesle A, Lim KS, SALSA Peer Leader Manual

(7th Edition) Western Sydney Local Health District. 2015

Enquiries about the SALSA kit should be directed to:

Clinical Associate Professor Smita Shah

Primary Health Care Education and Research Unit, Western Sydney Local Health District
Clinical Associate Professor, Faculty of Medicine, The University of Sydney

Mailing Address

PO Box 533, Westmead NSW 2145, Australia
Email: Smita.Shah@health.nsw.gov.au
T: +612 9845 6505 F: +612 9845 5310

The 7th edition of the *SALSA Peer Leader Manual* for the **SALSA** Program is funded by the Australian Government Department of Health.

© WSLHD 2015

This work is copyright. It may be reproduced in whole or in part for study or training purposes subject to the inclusion of an acknowledgement of the source. No commercial usage or sale of this publication is permitted. Reproduction for purposes other than those indicated above requires written permission from the author.

ISBN: 1 74080 145 8

Website: tinyurl.com/studentsaslifestyleactivists

Facebook: www.facebook.com/StudentsAsLifeStyleActivists

Instagram: @studentsaslifestyleactivists

NSW GOVERNMENT Health Western Sydney Local Health District

THE UNIVERSITY OF SYDNEY

Table of Contents

**Introduction**	**4**
How to use this manual	5
How will the SALSA program be run in schools?	6
Benefits of being a Peer Leader	7
The Do's and Don'ts of team work	8
Tips for managing a class	9
**Lesson 1: Food Choices**	**10**
Introduction	11
Ground Rules	12
Changing Chairs	13
Ryan's Goal Empowerment Activity	14
Lunch Box Lotto	16
**Lesson 2: Movement Matters**	**18**
What is Physical Activity?	19
Mapping Spaces	22
Charades: Guess the Problem	24
Let's Get Physical: Ultimate Frisbee	26
**Lesson 3: Healthy Lifestyle**	**27**
Energy Intake and Energy Used	28
Healthy Snacks	29
Cooking with your Family	32
Let's Get Physical: Ultimate Frisbee	32
**Lesson 4: SALSA Actions**	**35**
SALSA Quiz Show	36
Choose Your Own SMART Goal	38
School Action Plan	40
**Acknowledgements**	**41**

SALSA

Introduction for SALSA Peer Leaders

Welcome and congratulations on becoming a **SALSA** (Students As LifeStyle Activists) Peer Leader and Healthy Lifestyle Activist!

SALSA is a unique peer educational program designed to motivate high school students to increase physical activity, improve diet and lead a healthier lifestyle. The program uses a series of structured lessons, described in this manual, for trained **SALSA** Peer Leaders from Year 10 (15-16 year olds) to deliver to classes of Year 8 students (13-14 year olds).

As a **SALSA** Peer Leader and Healthy Lifestyle Activist, your role is to:

- Motivate and encourage Year 8 students to eat well and be more physically active
- Lead the activities for each lesson set out in this manual
- Model the healthy lifestyle practices you learn in the **SALSA** program
- Tell your friends and families about the benefits of leading a healthy lifestyle and support them to make changes

To ensure students listen and learn from you, it is very important to be confident and have a clear understanding of each **SALSA** activity. When talking to the class, stand tall, look up and speak clearly. Your teacher can help to oversee the management of the class.

Within each lesson, there are a number of activities to complete along with recommended times. You will need to finish these activities within this time limit so that the lesson does not run over time and also to ensure you cover the necessary information.

I wish to thank you for taking up this important leadership role to build a healthier school community.

Good luck and enjoy.

Smita Shah, *Clinical Associate Professor, School of Medicine, The University of Sydney and the Western Sydney Local Health District*

SALSA

How to use this manual

The SALSA Peer Leader Manual is a guide to help you prepare and deliver the lessons to the students. Each lesson is colour-coded and contains the information needed to run the activities.

WORKSHEETS:

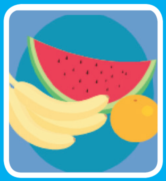

Lesson 1: Food Choices

- Introduction - 5 mins
- Ground rules - 5 mins
- Changing chairs - 10 mins
- Ryan's goal - 30 mins
- Lunch box lotto - 20 mins

Worksheets:

Ryan's Goal Video - Page 15

Lunch Box Lotto - Page 17

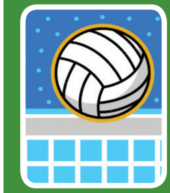

Lesson 2: Movement Matters

- What is physical activity? - 10 mins
- Mapping spaces - 15 mins
- Charades: guess the problem - 15 mins
- Let's get physical - 30 mins

Worksheets:

Physical Activity - Page 20, 21

Mapping Spaces - Page 23

Charades Key Cards - Page 25

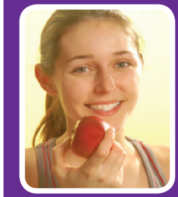

Lesson 3: Healthy Lifestyle

- Energy intake and energy used - 10 mins
- Healthy snacks - 10 mins
- Cooking with your family - 10 mins
- Let's get physical - 30 mins

Worksheets:

What is a Healthy Lifestyle - Page 30

Healthy Snacks - Page 31

Cooking Tips - Page 33, 34

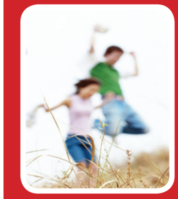

Lesson 4: SALSA Actions

- **SALSA** Quiz Show - 20 mins
- Create your own SMART Goal - 20 mins
- School Action Plan - 30 mins

Worksheets:

SALSA SMART Goal - Page 39

Symbols used in this manual

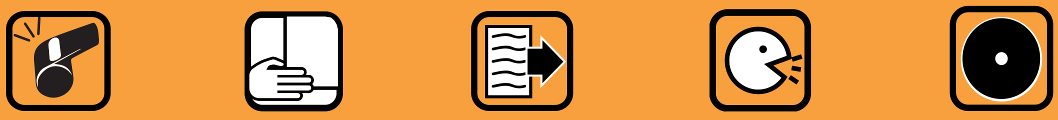

Rules | Worksheet | Instructions | What to say | DVD

How will the SALSA program be run in schools?

The SALSA program involves a 3-Step process as shown below.

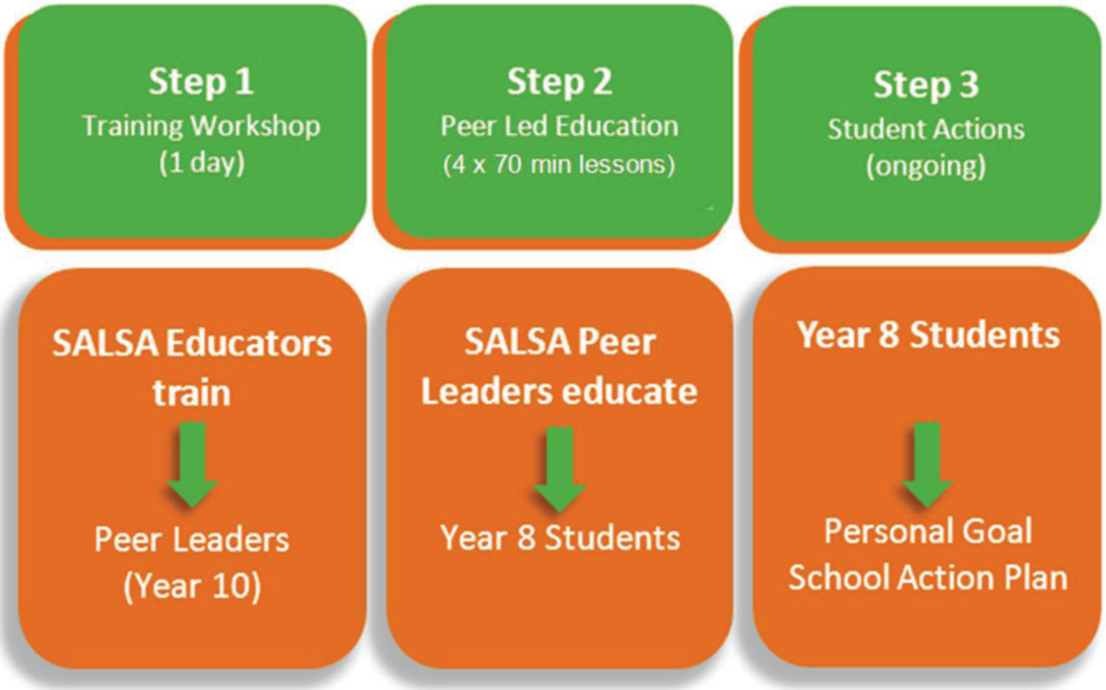

Step 1 **SALSA Educators** (university students) will train volunteer Year 10 students as SALSA Peer Leaders during a one-day workshop held at school.

The Peer Leaders will learn how to educate their younger peers about eating healthier foods and being physically active.

Step 2 **SALSA Peer Leaders** in teams of four will deliver 4 × 70 min lessons to Year 8 students using a DVD, games, and activities and this Peer Leader Manual as a guide.

The four lessons are: Food Choices, Movement Matters, Healthy Lifestyle and SALSA Actions.

Step 3 The **Class Teacher** together with the **Peer Leaders** will guide the Year 8 students to set personal healthy lifestyle goals and develop a School Action Plan to help support all students to maintain a healthy lifestyle.

Benefits of being a SALSA Peer Leader

As a SALSA **Peer Leader** you will:

- Learn new skills about successful teamwork
- Gain leadership skills
- Improve your communication and listening skills
- Gain knowledge and skills about healthy living
- Connect and make friends
- Have fun

Your contribution will motivate younger students to:

- Be more physically active
- Eat more vegetables and fruit
- Decrease time spent in sedentary activities
- Replace sugary drinks with water
- Increase awareness of services offered by local health providers
- Develop an action plan to maintain a healthy lifestyle

You can help to build a healthy school community!

SALSA Peer Leaders feedback:

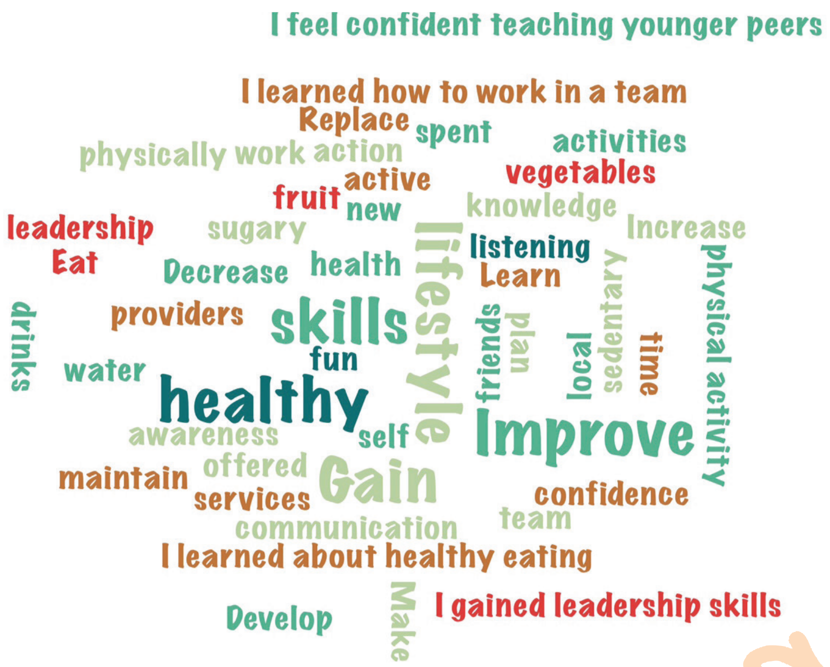

The Do's and Don'ts of team work

We suggest you work in groups of at least four to educate a class of Year 8 students

The benefits of working in a team include:

- You have the support of other team members
- Your buddies are there to help you deliver the lesson
- You can share the responsibility and workload
- You have someone to bounce off ideas
- You can make new friends

**Practice**	
**Do**	**Don't**
Have a practice session with your team before the lesson.	Just wing it and hope for the best.
**Be Organised**	
**Do**	**Don't**
Be clear who is doing what and when.	Just hope that you know what you are doing.
**Be Early**	
**Do**	**Don't**
Arrive early enough to set up the room in a semi-circle.	Be late.
**Keep to Time**	
**Do**	**Don't**
Start and end the lesson on time.<br>Complete all the activities.<br>Have a team member keep time.	Go over time.
**Seek Help from Your Team**	
**Do**	**Don't**
Have the other leaders or the teacher assist you if you need help to deliver your section or answer a difficult question.	Make up an answer to a question if you are not sure.

SALSA

Tips for managing a class

**Be prepared**	• Collect all handouts and equipment from your class teachers<br>• Meet with the team and go through the lesson in advance<br>• Practice is important
**Be early to set up the classroom**	• Be on time for lessons and set up the classroom in a semi-circle before the lesson starts
**Establish Ground Rules**	At the start of each lesson, establish Ground Rules to help you manage the class. Rules such as:<br>• Respect what others say even when you disagree<br>• Everyone should participate<br>• Listen to what other people are saying without interrupting<br>• No put downs<br>• Confidentiality
**If problems occur**	• Refer back to the Ground Rules<br>• Keep calm even if you are having difficulty maintaining the group's attention and have your team members help you<br>• If you have any issues with an individual student or with controlling the class, ask the teacher for help
**Conflict resolution**	• Try to sort things out so that everyone gets a fair go<br>• Try to avoid making the situation worse and keep your voice calm<br>• Address the problem in a positive way; have the "disruptive student" help with an activity, such as, writing on the board
**Follow the lesson plan**	• Remember to use this **Peer Leader Manual** when you run the four SALSA lessons

SALSA

LESSON 1 : Food Choices

Objective: Students will understand the benefits of eating healthy food and the importance of eating breakfast

Lesson Activities	Times	Resources
Introduction	5 mins	Name labels
Ground Rules	5 mins	Butcher's paper & markers
Changing Chairs	10 mins	Chairs
Ryan's Goal	30 mins	DVD, Ryan's Goal handouts
Lunch Box Lotto	20 mins	Lotto cards handouts

SALSA stands for Students As LifeStyle Activists.
A healthy lifetyle activist is someone who motivates and encourages others to eat well and be more physically active

In preparing for the lesson have you:

- Met with your peer leader team to discuss the lesson plan?
- Decided who will lead each of the activities?
- Checked the class list for student numbers?
- Checked that the dvd and player are available and working?
- Collected all the handouts from the teacher before the lesson?

SALSA FUN FACT

Green veggies are super good for you, because dark green vegetables like spinach, have a lot more Vitamin C than most fruits.

SALSA

Introduction (5 mins)

Have the class teacher introduce your team to the students and inform them that you will be conducting the four **SALSA** lessons

1. Introduce yourself by name. SALSA stands for Students As LifeStyle Acitvists
2. A healthy lifestyle activist is someone who motivates and encourages others to eat well and be more physically active
3. We will show you that being active and eating well is fun and easy to do

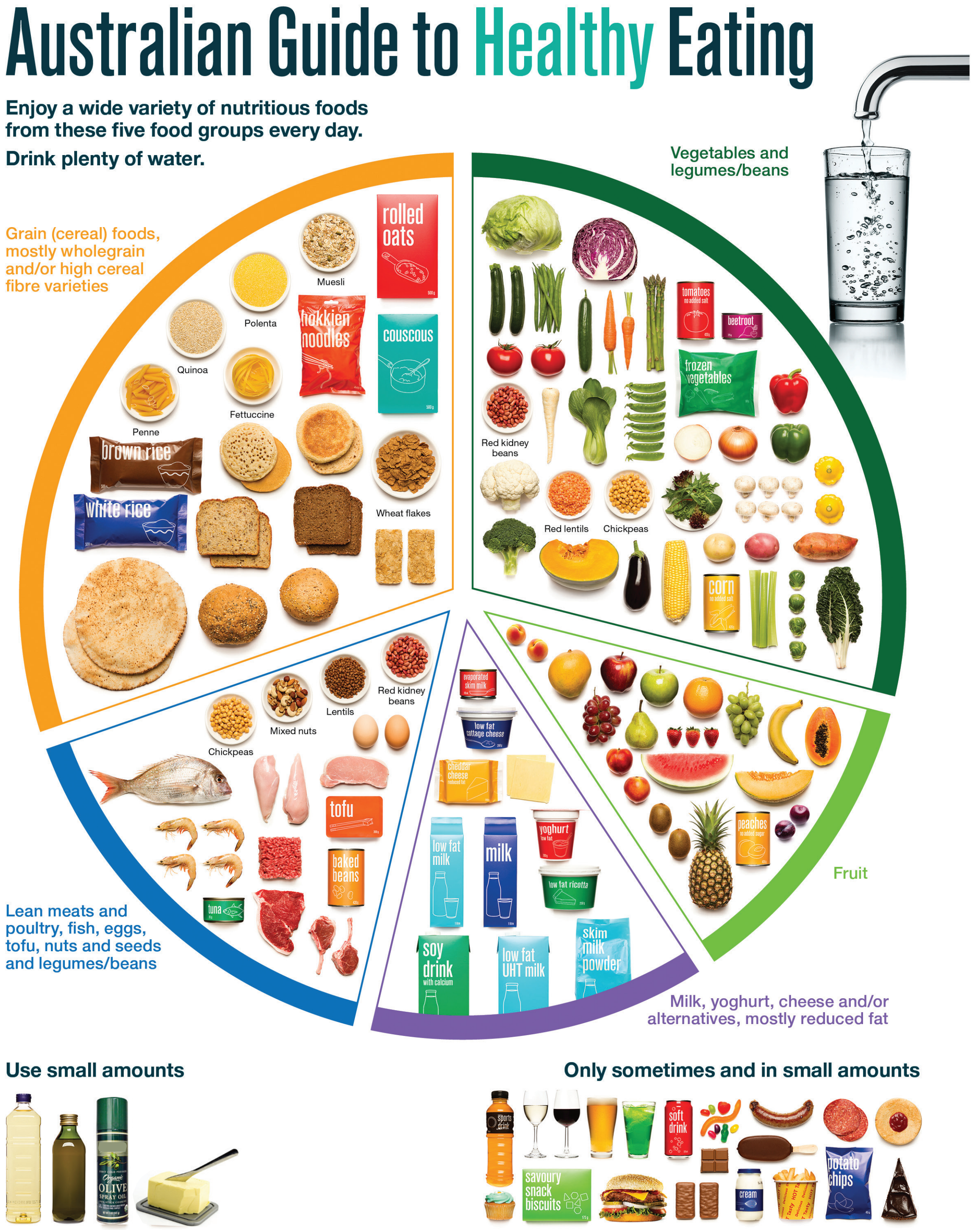

USEFUL LINK

For more information on the *Australian Guide to Healthy Eating* go to:

www.eatforhealth.gov.au/guidelines/australian-guide-healthy-eating

SALSA

Ground Rules (5 mins)

Purpose: Setting "Ground Rules" before you start will help your students stay focused and will help with class control so your lessons run smoothly

- For everyone to be able to learn and have fun, it is a good idea to have some Ground Rules

- Have the class decide on the Ground Rules
- Ask the class for suggestions and have a student write these on butchers paper
- Have the student read out the suggestions and ask for a show of hands to ensure that everyone is happy with the agreed rules
- Record the Ground Rules so you remember them for the next lesson
- Remind the group about confidentiality. Names stay in the group, but information can go out

Suggestions for Ground Rules:

✓ Respect each other

✓ One person speaks at a time

✓ Switch off mobile phones

✓ Confidentiality

✓ Have fun!

Changing Chairs (10 mins)

Purpose: To get the students to mingle and to discover what they have in common
To have fun so that students feel relaxed with you

Arrange the chairs in a circle, one less than the number of students in the group
Choose one Peer Leader to stand in the middle and start the game

- To start with, we are going to play a game called Changing Chairs
- One person stands in the middle, whilst others sit on chairs in a circle
- The person in the middle makes a statement, e.g. 'Hi, my name is Bob, everyone who came to school by bus please change seats'
- The person who is in the middle then moves quickly to get to a free seat
- The person who misses out on the seat then stands in the middle and makes another statement to get most people moving

Continue playing the game for about 5 minutes and then thank the students for their participation.

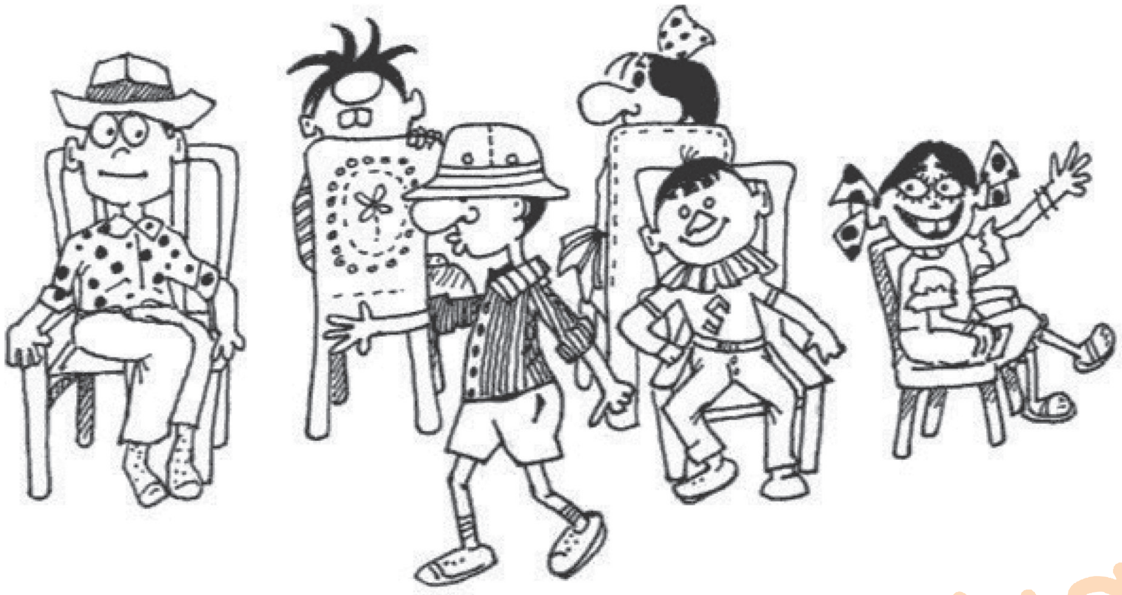

Ryan's Goal Empowerment Activity (30 mins)

Purpose: For students to discuss some of the lifestyle challanges faced by young people
This activity gives an opportunity for everyone to contribute to the discussion & offer solutions
It is important to involve all students and accept all answers

- Make 4 copies of the "Ryan's Goal Worksheet" handout, one for each group
- Provide butcher's paper for each group

- We will now watch a short movie called Ryan's Goal. It is a short drama about a 14 year old boy, Ryan, who sets himself a goal to get healthy
- We will discuss it in 4 groups once it has finished
- Play the DVD or YouTube on .... (15 min)

Group Discussion (5 min)

- Divide the students into 4 groups. One Peer Leader to join each group
- Have each group sit in a circle and go through the questions on the DVD discussion worksheet. Encourage all students to be involved
- Use the worksheet to record everyone's ideas (all answers need to be accepted)
- Ask a different student to read the questions and write the answers for each question

Presentation (10 min)

- Have each group present one key message from Ryan's Goal worksheet to the whole class
- Encourage the students to present their group's message in a creative way
- The message should be delivered in less than 2 minutes and can be presented on butchers paper
- Applaud each group's efforts

RYAN’S GOAL WORKSHEET

1. What do you see as the key messages in Ryan’s Goal?

2. How did Ryan feel about his lifestyle habits at the start of the movie?

3. Share similar experiences from your own lives.

4. Why do you think some young people skip breakfast?

5. What is your take home message from Ryan's Goal?

SALSA

Lunch Box Lotto (20 mins)

Purpose: To inform students about different healthy options for lunch

- Distribute a Lunch Box Lotto worksheet to each student

- For this game, it is best if each Peer Leader has a designated role. Two Peer Leaders give a worksheet to each student. One Peer Leader to reads out the rules and the other Peer Leader to check that everyone knows what they are doing

- We are now going to play a game called Lunch Box Lotto

- Each person needs to pack their imaginary lunch order from the '*Good Food Canteen Menu*' shown on the worksheet

- Fill each box on your card with one item from the list (wait 2 minutes for them to write down their food choices)

- I will now call out items from the menu in a random order. If the item is in your lunch box, cross it out

- The first person with all words crossed out, calls out **SALSA!** to win the game

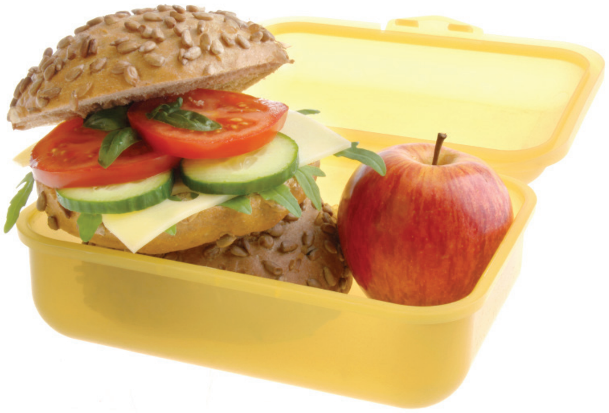

SALSA

LUNCH BOX LOTTO WORKSHEET

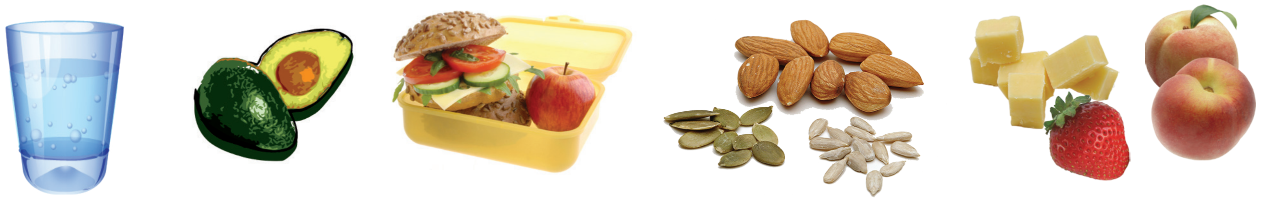

1. Select foods for your lunch box from the Good Food Canteen Menu below
2. Write one item in each box
3. A Peer Leader will read out items from the canteen menu in random order
4. Cross off the items in your lunch box as they are called out
5. The first person with all their items crossed out, calls out "**SALSA!**" and wins the game

GOOD FOOD CANTEEN MENU

Bread	Snack	Drink
Wholemeal, Turkish, Lavash, Lebanese, Pita, Raisin, Wholegrain, Rye	Yoghurt, Frozen Yoghurt, Dried Fruit, Cheese & Crackers, Nuts	Water, Plain milk, Tea
**Sandwich Fillings**	**Fruit & Veggies**	**Other**
Lean meats, Egg, Cheese, Carrot, Lettuce, Tomato, Tuna, Cucumber, Avocado	Apple, Orange, Banana, Peach, Mango, Pear, Grapes, Carrot sticks, Celery sticks, Salad	Minestrone soup, Pumpkin soup, Rice, Pasta salad, Chicken soup

USEFUL LINK
www.goforyourlife.vic.gov.au/hav/articles.nsf/html/index.html
Play this game online to create a balanced lunchbox and receive feedback

LESSON 2: Movement Matters

Objective: For students to understand the importance of regular physical activity

Lesson Activities	Times	Resources
What is Physical Activity?	10 mins	Physical Activity key cards<br>Scissors
Mapping Spaces	15 mins	A3 maps, dot stickers<br>Mapping Spaces Worksheet
Charades	15 mins	Charade key cards
Let's Get Physical	30 mins	Frisbee, witches hats, coloured bibs or sashes

In preparing for the lesson have you:

- Met with your peer leader team to discuss the lesson plan?
- Collected all the handouts from the teacher before the lesson?
- Met with your teacher to organise the frisbee activity?
- Cut out the Physical Activity cards?
- Cut out the Charades Key cards?

SALSA FUN FACT

Did you know depressed people can improve their symptoms by as much as 47 % with regular aerobic exercise?

USEFUL LINK

www.cdc.gov/bam/activity/index.html
Helpful information on and suggestions for physical activity

SALSA

What is physical activity? (10 mins)

Purpose: For students to understand the difference between planned, incidental & sedentary activities

You will need: Scissors, Physical Activity Cards and worksheet

Review the ground rules to maintain control of the group

- Make four copies of the 'Physical Activity Worksheet' and 'Physical Activity Cards'
- Cut out the cards from the worksheet and supply a set of cards to each of the teams
- Divide the students into 4 groups

- Ask the group "Why do you think we should be more active?" Allow students to respond
- Summarise by saying "You see there are many benefits in being more active. These include having fun, feeling good, spending time with friends and so on"

- We are now going to play a game which looks at the difference between planned, incidental and sedentary activities

- We are going to give you a set of cards which are examples of these different forms of activities
- Look at the definitions of each of these activities on the worksheet and place the cards accordingly under the correct headings
- Once everyone in the team agrees where the cards should go, call out '**SALSA**'
- The first group to have all cards under the correct headings wins

Why be active?

- Feel better and more energetic
- Improve your fitness and strength
- Make new friends
- Sleep better
- It's fun!

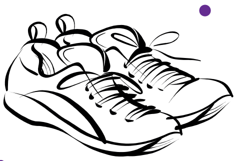

PHYSICAL ACTIVITY CARDS

1. Make 4 copies of the Physical Activity Cards sheet
2. Cut out the cards and handout a set to each group

PLANNED ACTIVITY	INCIDENTAL ACTIVITY	SEDENTARY ACTIVITY
Running	Taking the stairs	Watching TV
Dance class	Walking to the bus stop	Computer games
Swimming lessons	Walking the dog	Sitting and texting friends
Riding a bike	Dancing around to great music	Surfing the internet
Netball	Vacuuming	Eating meals

"Encouraging children and young people to give up even 30 minutes of daily small screen recreation in exchange for physical activity can make a genuine difference to their health and well-being".

www.facebook.com/StudentsAsLifestyleActivists

SALSA

PHYSICAL ACTIVITY WORKSHEET

PLANNED ACTIVITY	INCIDENTAL ACTIVITY	SEDENTARY ACTIVITY
When your body is doing activities that require the use of more energy at a moderate to high level.	Movements that are a part of everyday life. These are important to stay healthy as well.	Actions that do not use much energy and usually occur while sitting.

SALSA

Mapping Spaces (15 mins)

Purpose: To identify a range of facilities where you can be active in your local area

You will need: A3 maps of your local area and Dot stickers

- Make four copies of the "Mapping Spaces" handouts. Give one handout and one map to each team

- Part of staying healthy is keeping active. Physical activity can mean a lot of things, not just going to the gym or playing sports
- There are many places you can do physical activity
- What we will do now is find places in our local area where we can be active

- Keep the students in 4 groups
- For Mapping Spaces, lay out the A3 maps you have of the local area
- Have students place a dot sticker on the map to indicate areas where they can play or do physical activity
- Next, discuss other ways they might use these spaces for physical activity
- Have them answer the two questions from the Mapping Spaces Activity sheet

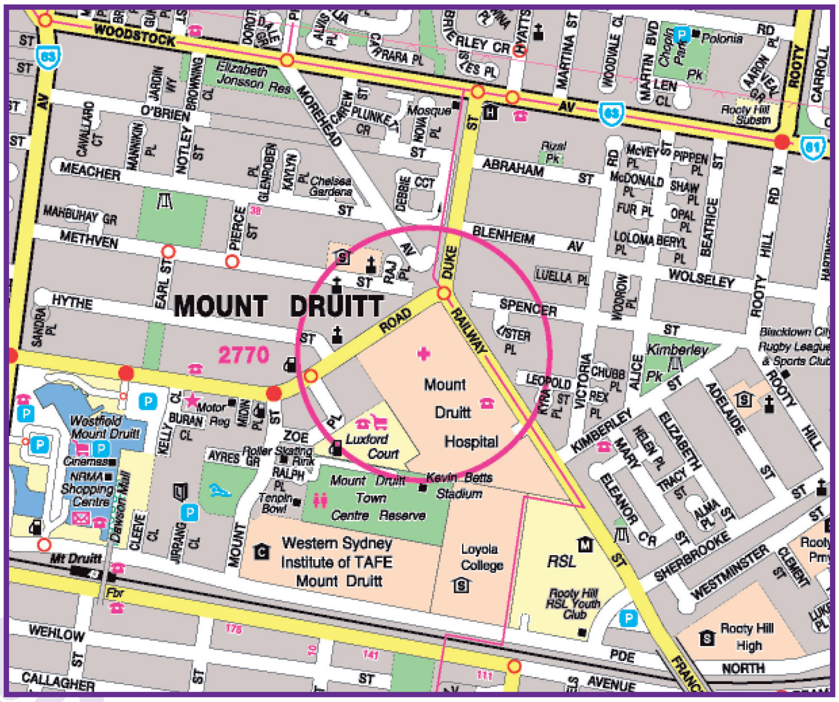

MAPPING SPACES WORKSHEET

1. Where in your local area can you participate in activities that you are interested in?

Sport or physical activity	Where?

2. How can we turn a sedentary activity into an incidental or a planned activity?

**Sedentary activity**<br>*E.g. Playing computer games*	**Incidental or planned activity**<br>*E.g. Playing outside with friends*

SALSA

Charades: Guess the problem (15 mins)

Purpose: To increase awareness about the services available to manage common health problems

- Make a copy of the “Charade Key Cards” and cut them out

- Health problems can arise at any time in your life
- There are many people and places in the community you can go to for assistance with any health problems you may have
- It is important to remember not to be embarrassed to seek help. It is the job of health professionals to help you

- Divide the class into four groups and give each group a health card
- Give each group a specific key card with a health problem and have them decide on a service that can be used for the problem
- Have each group act out this health problem as a charade to the class
- The class needs to guess what the problem is and who they should see

Some health issues and where you can go for help are listed below:

Problems	Who can help
Skin problems	General practitioner (GP)
Depression	GP, School Counsellor, Social Worker, Psychologist
Asthma	GP, School First Aid officer
Quit smoking	GP, Quitline or website
Toothache	Dentist
Problems at home	GP, School Counsellor, Teacher, Family member

CHARADES KEY CARDS

SKIN PROBLEMS<br>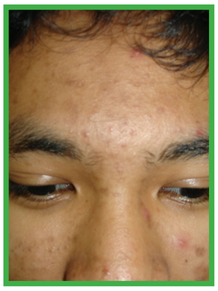	ASTHMA<br>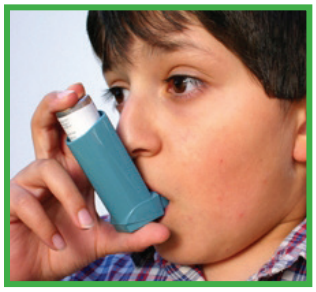
TOOTHACHE<br>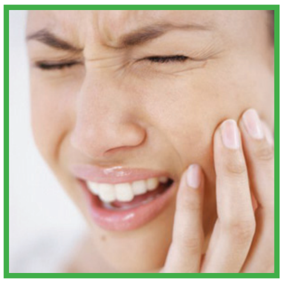	QUIT SMOKING<br>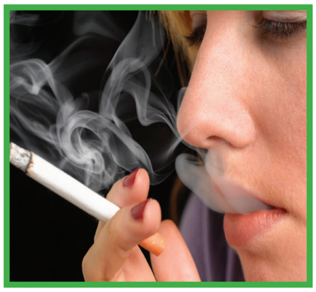
DEPRESSION<br>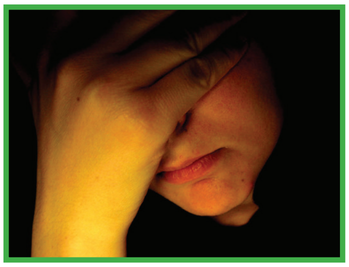	PROBLEMS AT HOME<br>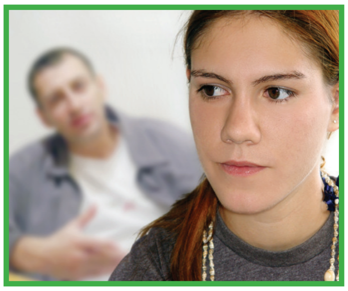

SALSA

Let's Get Physical: Ultimate Frisbee (30 mins)

Purpose: To give students the opportunity to participate in a form of physical activity that is simple, fun and easy to do. The classroom teacher needs to deliver this activity

You will need: A frisbee, witches hats and coloured bibs or sashes for team identity

- Leave the classroom and move to a suitable outdoor PE area to play
- Split the group into 2 teams of even numbers
- Set up the playing area using cones
- The size of the field will depend on the amount of playing area in the school
- An approximate size would be that of a touch football field (small section of a full size soccer/football field)
- Play Ultimate Frisbee using instructions set out below

Rules of Ultimate Frisbee

Initiate play
Each point begins with both teams lining up on the front of the respective `end zone' line (try line). The defence throws the disc to the offence.
**Scoring**
Each time the offence completes a pass in the defence's end zone, the offence scores a point. Play is initiated after each score.
**Movement of the disc**
The disc may be advanced in any direction by completing a pass to a team mate. Players may not run with the disc. The person with the disc (`thrower') has 10 seconds to throw the disc. The defender guarding the thrower (`marker') counts down the 10 seconds.
**Change of possession**
When a pass is not completed (e.g. out of bounds, drop, block, interception), the defence immediately takes possession of the disc and becomes the offense.
**Non-contact**
No physical contact is allowed between players. A foul occurs when contact is made.
**Fouls**
When a player initiates contact on another player a foul occurs. When a foul disrupts possession, the play resumes as if the possession was retained. If the player committing the foul disagrees with the foul call, the play is redone.

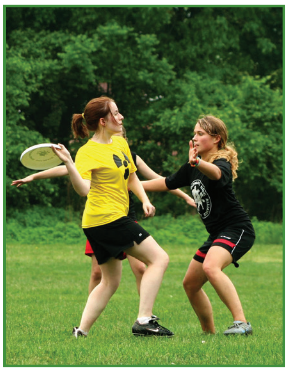

Please note:
Teachers will need to be prepared with an alternative indoor activity if wet weather prohibits outdoor play

SALSA

LESSON 3: Healthy Lifestyle

Objective: Students will gain knowledge and skills to make more informed healthy lifestyle choices

Lesson Activities	Times	Resources
Energy Intake and Energy Used	15 mins	What is a Healthy Lifestyle? Worksheet
Healthy Snacks	15 mins	Healthy Snacks Worksheet
Cooking Tips	15 mins	Healthy Ideas for Home Worksheet
Let's Get Physical	30 mins	Frisbee, cones, coloured bibs or sashes

In preparing for the lesson have you:

- Met with your peer leader team to discuss the lesson plan?
- Collected all the handouts from the teacher before the lesson?
- Met with your teacher to organise the Frisbee activity?
- Collected the equipment needed for Frisbee?

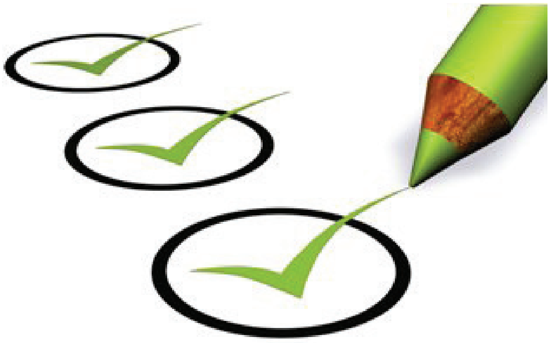

SALSA FUN FACT

Did you know that students who eat breakfast perform better in the classroom and on the playground, with better concentration, problem-solving skills, and eye-hand coordination?

Energy Intake and Energy Used (10 mins)

Purpose: To show students that for a healthy mind and body, you need a healthy lifestyle

Rules

Review the ground rules to maintain control of the group

- Have the students stand in one big circle
- “What does healthy lifestyle mean to you?" Allow the students to respond
- Ask students to mark the place they are standing, by placing an item at their feet, e.g. a pen
- Have one Peer Leader give examples of 'Energy Intake' and 'Energy Used' - see list for examples
- The students will move **out** if they think the example is 'Energy Intake' and **in** if they think it is 'Energy Used'
- Make sure you give the same amount of 'Energy Intake' and 'Energy Used' examples, so that the students end up in their original position. Ask the students what they could do if they end up in a different position than where they started
- At the end of the activity, give each student a 'What is a healthy lifestyle?' Worksheet to complete and discuss as a group

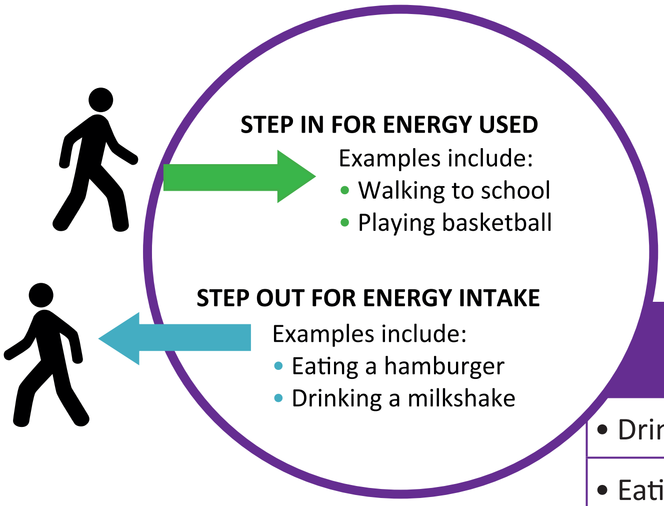

Some reasons to lead a healthy lifestyle are:

✓ You will feel better and have more energy
✓ Improves concentration at school
✓ Sleep better

Examples of Energy Intake	Examples of Energy Used
• Drinking a glass of milk	• Going for a run
• Eating a hamburger	• Cycling to a friend's house
• Eating a piece of fruit	• Doing a cross-fit class
• Having a bag of chips	• Playing a game of ultimate frisbee
• Drinking a glass of juice	• Walking to the shops

SALSA

Healthy Snacks (10 mins)

Purpose: To show students how to include healthy snacks in their diet and how to avoid snacks high in fat, salt and sugar

- It is important to identify healthy snacks and work out ways to make sure they are part of your eating habits

- Divide the class into four groups
- Provide the students with a 'Healthy Snacks' worksheet
- Have each student complete the questions on the worksheet
- Have the students write down their answers on their sheet
- Encourage the students to take the worksheet home and share their ideas with their family

USEFUL LINK

Read about 5 ways to lead a healthy lifestyle:
www.healthykids.nsw.gov.au/home/fact-sheets/5-ways-to-a-healthy-lifestyle.aspx

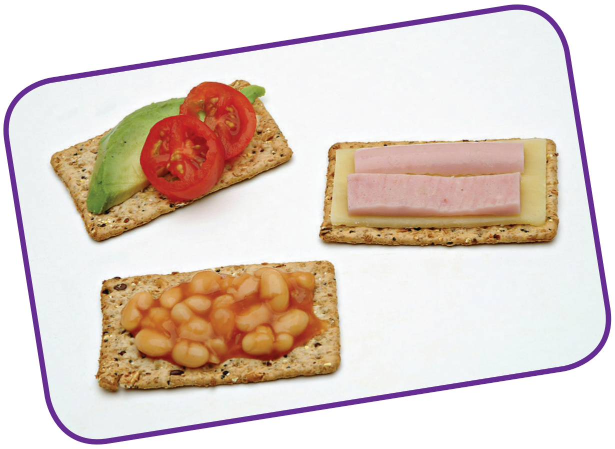

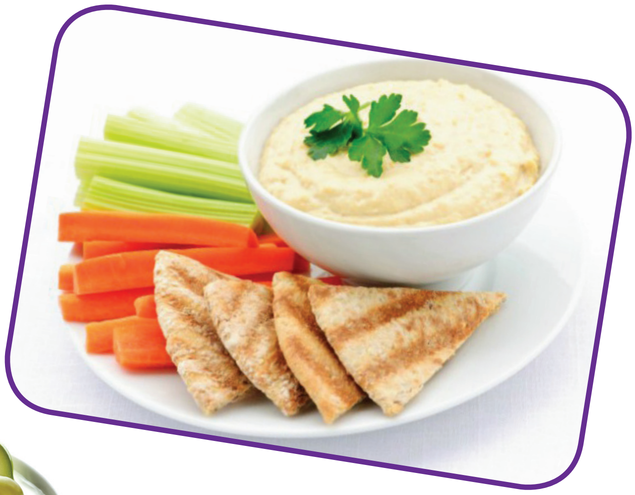

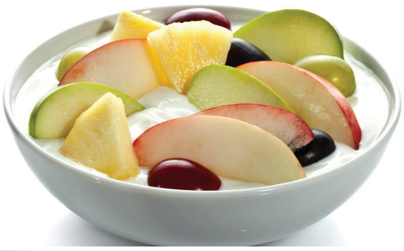

SALSA

WHAT IS A HEALTHY LIFESTYLE?

EAT WELL, DRINK WELL AND BE FIT!

Fill in the blanks

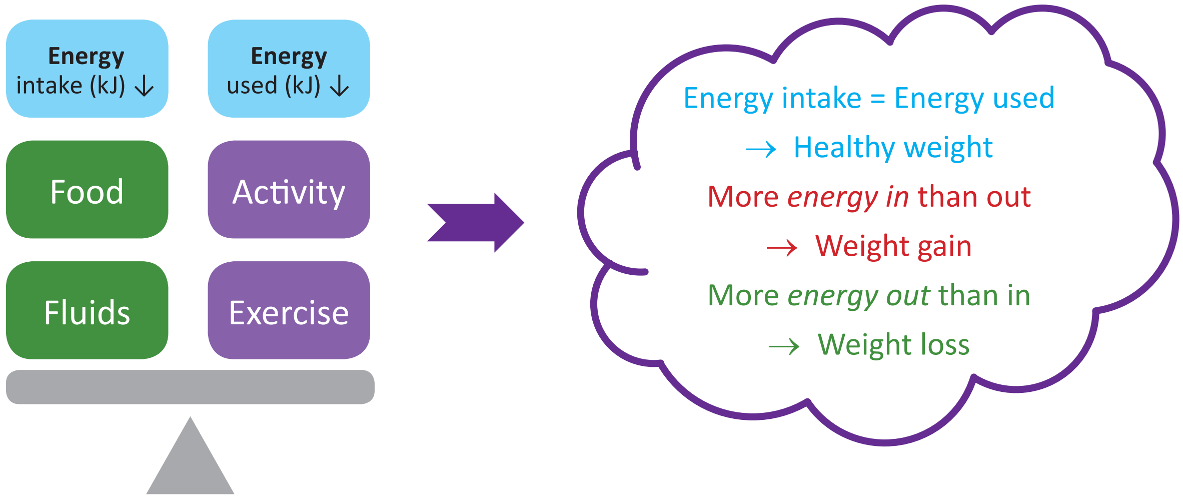

Energy intake

Eat more f_____ and v__________

- The key is to pick lots of different colours for nutrients
- Go for __ pieces of fruit and at least __ serves of vegetables every day

Drink plenty of w_____

- Water is the best way to quench your thirst
- A 600mL bottle of soft drink (Cola) has 16 teaspoons of s_____ in it!!
- Sugary drinks, soft drinks and fruit juice can lead to t_____ decay and weight gain

Eat Breakfast daily

- B_________ is the most important meal of the day
- Breakfast feeds your body and mind with the nutrients and energy to function well throughout the day

Energy used

Do some moderate physical activity

- Kicking a ball around with friends
- ______________________________
- ______________________________
- ______________________________

Do some vigorous physical activity

- Netball
- ______________________________
- ______________________________
- ______________________________
- Running

Limit the time you spend:

- Watching television
- Playing video games
- Using social media
- Using the computer

HEALTHY SNACKS

Snacks can be part of a healthy diet, however, some can be high in energy, sugar and salt.

What are some reasons young people might <u>not</u> eat healthy snacks?

....

....

....

....

What could be done to change this?

....

....

....

....

To the right are some examples of healthy snacks. Add to this list by including your own healthy snack ideas.

SALSA TIP: ***Sometimes people snack because they are actually thirsty. Try drinking water before deciding to have a snack.***

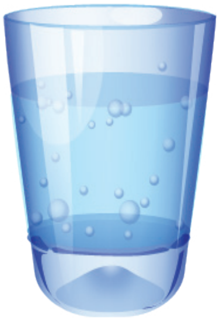

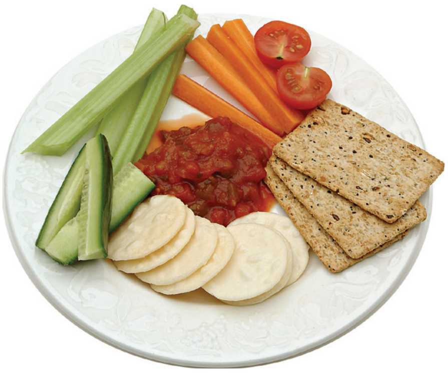

- Fresh fruit
- Vegetable sticks with hummus
- Vita-Weat topped with cottage cheese, Vegemite or tomato
- Rice crackers or baked wheat pretzels
- A handful of raw nuts and seeds
- Plain yoghurt or milk
- Sushi with tuna or vegetables
- Small can of baked beans, canned tuna or salmon
- Toasted cheese and tomato sandwich
- Edamame (soya beans)
- ______________________________
- ______________________________
- ______________________________

USEFUL LINK

www.cdc.gov/bam/nutrition/index.html
Helpful information on and suggestions for healthy snacks

SALSA

Cooking with your family (10 mins)

Purpose: To learn easy ways to make healthy foods
Students should be encouraged to share the cooking tips with their families and friends

- Copy "Cooking Tips" and "Healthy Ideas for Home Cooking" handouts for each student
- Hand out the worksheets to the 4 groups
- Have one student read out each cooking tip from handout
- Explain the two columns of the 'Healthy Ideas for Home Cooking' worksheet
- Ask the students to decide on 3 cooking tips they would like to try at home
- Students fill out the '3 easy cooking tips' and 'one healthy meal' on their handout

Cooking Tips

Increase vegetables and fruit

- Choose a variety of different coloured vegetables each meal
- Add fruit to any desserts you make
- Eat fresh or frozen vegetables and fruit

Increase fibre

- Use wholemeal/wholegrain varieties e.g. brown rice, wholemeal bread and pasta

Reduce fat

- Use small amounts of oil in cooking and salads
- Trim excess fat from meats
- Grill instead of frying
- Stir-fry, steam or microwave your vegetables

Limit Salt

- Limit the amount of salt added during cooking and at the table
- Add herbs and spices for more flavour

When serving a balanced meal ensure that:

- Vegetables cover half the plate
- Carbohydrates cover a quarter of the plate
- Lean meat, fish or plant based protein covers the last quarter of the plate

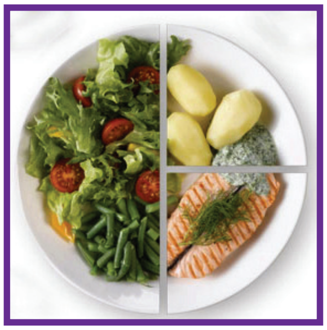

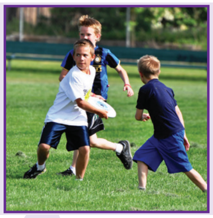

Let's Get Physical - Ultimate Frisbee (30 mins)

See page 26 for instructions to play ***Ultimate Frisbee***

SALSA

Cooking Tips

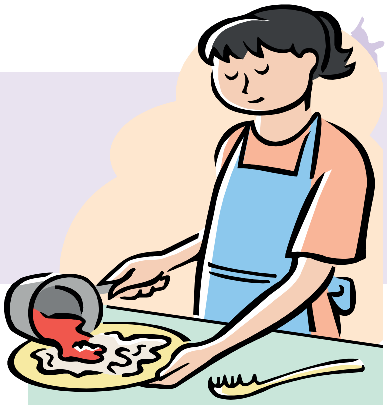

Three easy cooking tips I will share with my family:

1. ....
2. ....
3. ....

One healthy meal I can share with my family:

....

....

....

My SALSA Lunch Box

Using the cooking tips, make and bring a healthy lunch,

on the day of our next SALSA class

USEFUL LINK

Useful websites for current health information:

One of Australia's National Health websites:
www.swapit.gov.au

A service for youth who are at risk of harm and to help them realise their potential:
www.youthoutreach.org.au/

A healthy lifestyle website specifically for children and teenagers:
www.healthykids.nsw.gov.au/kids-teens.aspx

Balanced daily meals and snacks game:
www.eatforhealth.gov.au/game/index.html

Healthy and fun recipes for kids and teenagers:
www.gofor2and5.com.au/KidsOnly/KidsintheKitchen/tabid/208/Default.aspx

Kids and teenagers healthy living:
www.schn.health.nsw.gov.au/kids-and-teenagers/teenagers/healthy-living

Tips and ideas from the Heart Foundation:
www.rch.org.au/cah/resources/Links/

HEALTHY IDEAS FOR HOME COOKING

FOOD	HEALTHIER TAKE-AWAY OPTION	HOME OPTION
Pizza 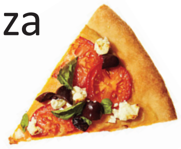	• Less cheese<br>• Limit the amount of meat on the pizza and add extra vegetables<br>• Thin crust	• Lebanese bread with a variety of vegetables, mozzarella cheese and only one meat
Chips 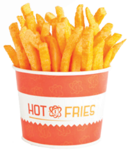	• Thicker chips<br>• Baked potato	• Oven fries<br>• Potatoes tossed in olive oil and baked on a tray in the oven<br>• Sweet potato chips
Asian Food 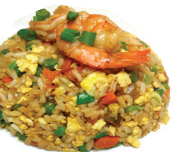	• Steamed instead of fried rice<br>• Stir fries rather than curries<br>• Steamed entrees rather than deep fried	• For a quick stir fry at home, add lean meat, beans or tofu, fresh vegetables<br>• Serve with steamed rice<br>• Add frozen vegetables as a quick, healthy alternative
Wraps 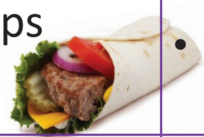	• Less meat and more vegetables e.g. tabbouleh	• Home made wraps with lean meat and salad
Chicken 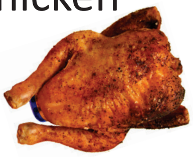	• Remove the skin from the chicken<br>• Have it without chips	• Home made chicken burger with grilled chicken and plenty of salad and vegetables
Burgers 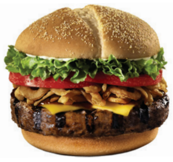	• Go for burgers without the extras e.g. bacon and cheese<br>• No mayonnaise on the burger<br>• Have it without chips	• Use low fat mince to make burgers at home<br>• Add plenty of salad to your burger e.g. lettuce, tomato, onion, beetroot, avocado
Pasta 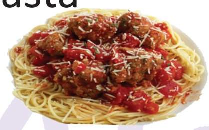	• Go for tomato based sauce rather than cream based	• Use lean mince and diced tomatoes<br>• Add herbs for flavour<br>• Wholegrain pasta

SALSA

Lesson 4: SALSA Actions

Objective: Students will apply the knowledge learnt in previous lessons to maintain a healthy lifestyle

Lesson Activities	Times	Resources
SALSA Quiz Show	20 mins	
Create your own SMART Goal	20 mins	SMART Goal handout
School Action Plan	30 mins	Butchers paper

In preparing for the lesson have you:

- Met with your Peer Leader team to discuss the lesson plan?
- Collected all the handouts from the teacher before the lesson?

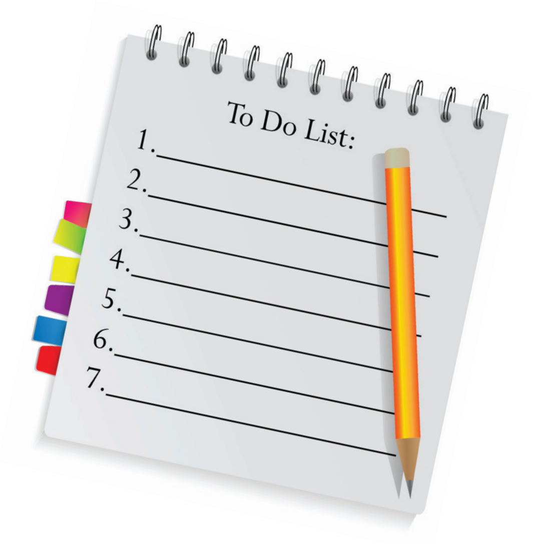

SALSA FUN FACT

Did you know that you burn more kilojoules sleeping than you do watching television?

SALSA Quiz Show (20 mins)

Purpose: To revise key facts learnt about healthy eating and physical activity

Review the ground rules to maintain control of the group

- Divide the students into 2 teams
- Have each team come up with a team name
- Introduce the Quiz Show using the dialogue below
- Have each team nominate one person to keep score
- One Peer Leader reads each question
- Wait for the correct response from the teams

- I will ask 10 questions that need to be answered by a student in the team
- A second Peer Leader reads out the correct answer
- The student who puts their hand up first gets to answer the question
- If they are correct, they receive 10 points; if they are incorrect, they lose 10 points
- If the other team can give a better answer, then they get 10 bonus points
- Each student can only answer one question, so everybody can have a go
- Teams must give a reason when answering true or false questions

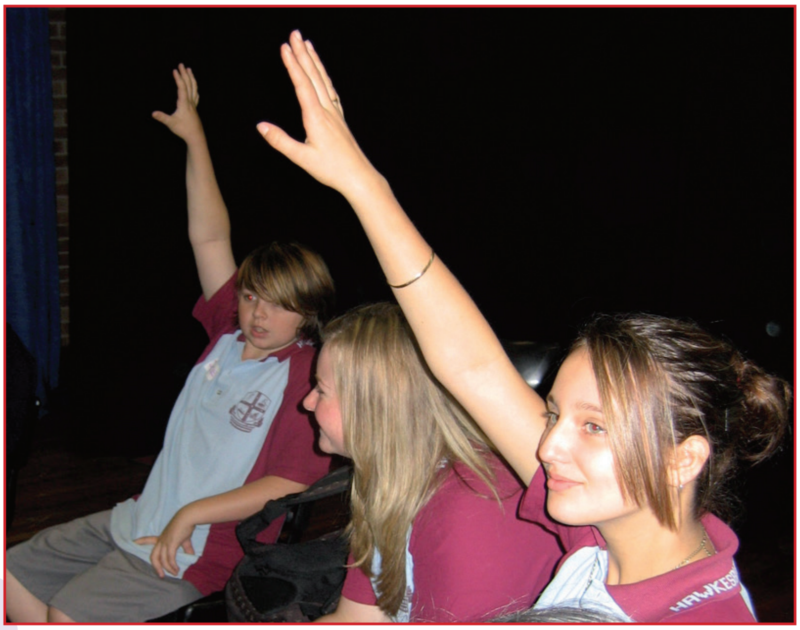

SALSA

SALSA Quiz Show Questions

1. ***What does SALSA stand for?***
Students As LifeStyle Activists

2. ***How much physical activity should adolescents be doing?***

a) Half an hour of moderate physical activity 3 times a week

b) Half an hour of moderate physical activity every day

c) At least 1 hour of moderate to vigorous physical activity every day

3. ***Drinking bottled fruit juice is as good as eating fruit. True or False?***

FALSE: Bottled fruit juice contains large amounts of sugar (similar to the amounts in soft drinks).

It does not have the same nutritional value as fresh fruit, and provides less fibre than whole fruit

4. ***The food you eat affects your mood and energy levels. True or False?***

TRUE: Foods high in sugar (lollies, soft drinks, fruit juices) give you a quick burst of energy but this does not last long and can leave you feeling flat and tired later

When you eat a range of nutritional foods you are able to think more clearly and feel better.

5. ***It is OK if you miss breakfast provided that you eat well during lunch and dinner. True or False?***

FALSE: There are approximately 12 hours between eating your dinner and waking up. Skipping breakfast can often leave you feeling fuzzy or unable to concentrate. This is because your brain is lacking energy. Eating breakfast helps to kick start your metabolism.

6. ***Tap water is the best drink for your teeth and general health. True or false?***

TRUE: Only tap water has protective Flouride for your teeth. Water keeps you hydrated without the added kilojoules.

7. ***Your local GP will only assist you with physical problems. True or false?***

FALSE: You can seek help or advice from your GP for a range of emotional, social or physical issues.

8. ***Approximately how many teaspoons of sugar are there in a 600ml bottle of coke?***

a) 13

b) 16

c) 7

9. ***Energy drinks are good for your health.***

FALSE: Like soft drinks, energy drinks are high in kilojoules. Additionally, energy drinks often contain stimulants like caffeine and guarana which can be bad for your heart and blood pressure.

10. ***It is recommended that teenagers limit recreational screen time e.g. social media, texting, TV and PC use to:***

a) 4 hours maximum

b) 3 hours maximum

c) 2 hours maximum

SALSA

Create your own SMART Goal (20 mins)

Purpose: For students to set a SMART Goal

- Each letter in SMART will help guide you when making your goal
- Shown below is what each letter stands for and what you should consider when making your very own goal. Examples are given to help you

SMART Goal Action Plan

One Peer Leader to facilitate each group

Ask: "Who can remember the 7 key messages from Ryan's Goal?"

1. Reduce time spent watching TV and playing video games
2. Have a healthy breakfast everyday
3. Reduce soft drinks or sugary fruit juices
4. Take every opportunity to be active, e.g. walk to school
5. Eat regular healthy meals
6. Reduce "junk food"
7. Be active after school e.g. join a sports club

- Read through the SMART Goal table, and have each student come up with one short term SMART goal that they would like to attempt and fill out their own SMART Goal Chart.
- Within your group, ask each student how they plan to achieve their goal.

	×Example needs work	✓Better example
**Specific:**	Be healthier	Cut down on the number of chocolate bars I eat per week
**Measureable:**	Eat less chocolate	Have only 2 chocolate bars a week
**Achievable:**	Never eat chocolate again	Have only 2 chocolate bars a week
**Relevant:**	Give up eating biscuits (when you don't like or never eat biscuits)	Reduce the number of chocolate bars I eat
**Time-limited:**	Have only 10 chocolate bars this year	Have only 2 chocolate bars this week

USEFUL LINK

To re-watch Ryan's Goal at home visit:
www.youtube.com/watch?v=v_qbKbJ1sBg

SALSA

Put this page up some where to help you remember your goal

SALSA

My Healthy Lifestyle SMART Goal is:

____

____

____

To be achieved by:

____

(Date)

Tips for reaching your smart goal

- Set yourself a doable goal
- Ask for support
- Set a time limit
- Make it part of your routine

Specific
Measureable
Achievable
Relevant
Time-limited

SALSA

SALSA

School Action Plan (30 mins)

Purpose: For students to apply what they have learned into action
This activity should involve the classroom teacher
Butchers paper should be provided for brainstorming

Over the past weeks we have spoken about ways to lead a healthy lifestyle. We have learnt about eating foods that are good for you and the importance of staying fit. In groups we will now decide on some key messages and write action plans to help make a difference in our school.

- In four groups, discuss ideas for a School Action Plan
- Each group must chose one Action Plan
- Have your teacher check that the plan could be done in your school
- Discuss what steps are needed to achieve this action. This should include:

1. **The aim of the Action Plan**
2. **Who will be involved?**
3. **Which teachers do you need to speak to about it?**
4. **The role of each person involved and what they will need to do**
5. **When will it be done?**
6. **Where will it be done?**

- Write down the steps on paper, present your plan to the class, and have the class decide on at least one 'School Action Plan' that can be done at school

For example
Action: Starting a lunch time footy group
Steps: - Form a group
- Put a notice in the school newsletter
- Bring a ball
- Find a teacher to supervise

At the end of this lesson, peer leaders should hand each class's action plan to the teacher, the class teacher can follow up this activity in future lessons

Examples of School Action Plans:

- More fruit and affordable healthy snacks for sale at the canteen
- Start a school vegetable garden
- Create a cooking app

SALSA

Acknowledgements

Authors:

Associate Professor Smita Shah, Amy Andrew, Crystal Hillery, Andrew Pesle, Dr Kean-Seng Lim.

Contibutors:

Associate Professor Nihaya Al Sheyab, Mara Cvejic, Hayley Forbes, Dr Ralf Itzwerth, Dr Corinne Patching, Dr Vanessa Shrewsbury.

This program arose from a need identified in the community and resulted in collaborative action. We thank the many individuals and organisations that have contributed to the development of the SALSA program and its resources.

Partners:

- Blacktown Medical Practitioners Association
- Mt Druitt Medical Practitioners Association
- Rooty Hill High School
- The University of Sydney
- Western Sydney Local Health District

Funding 2013-2015:

- Australian Government Department of Health

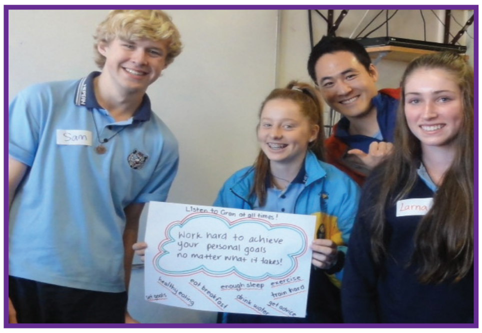

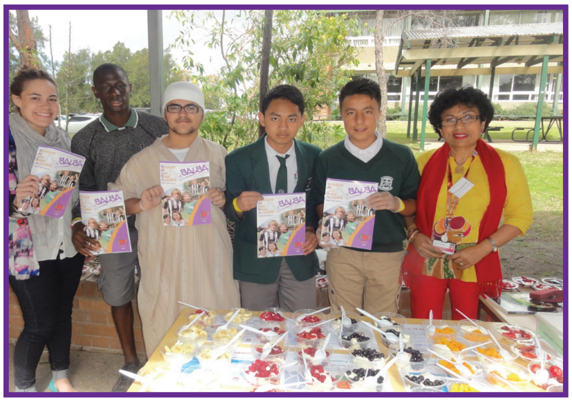

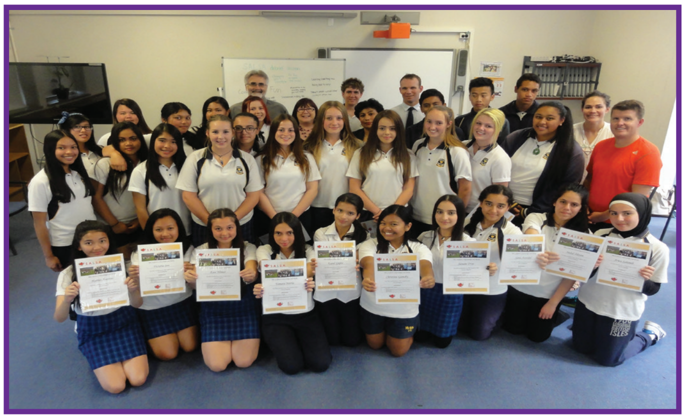

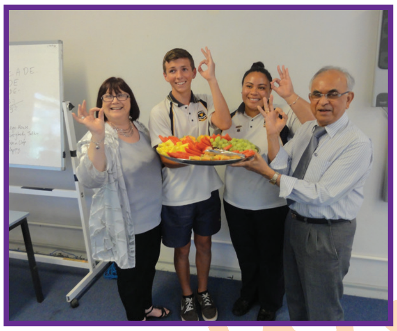

SALSA

NOTES

NOTES

SALSA

SALSA

SALSA

SALSA
